# Calprotectin and Receptor for Advanced Glycation End Products as a Potential Biomarker in Abdominal Aortic Aneurysm

**DOI:** 10.3390/jcm9040927

**Published:** 2020-03-28

**Authors:** Willy Hauzer, Wojciech Witkiewicz, Jan Gnus

**Affiliations:** 1 Department of Vascular Surgery, Regional Specialist Hospital, 51-124 Wroclaw, Poland; witkiewicz@wssk.wroc.pl; 2 Research and Development Center, Regional Specialist Hospital, 51-124 Wroclaw, Poland; 3 Department of Physiotherapy, Wroclaw Medical University, 50-355 Wroclaw, Poland; jan.ngus@umed.wroc.pl

**Keywords:** abdominal aortic aneurysm, receptor for advanced glycation end products, calprotectin, biomarkers

## Abstract

Experiments conducted in recent years on animals and research works worldwide show a linkage between calprotectin and occurrence and development of the abdominal aortic aneurysm (AAA). Additionally, a correlation between the level of the receptor for advanced glycation end products (RAGE) and the diameter of the abdominal aorta was found. The purpose of this study was to investigate whether calprotectin and the RAGE plasma level may be a biomarker of human AAA occurrence. We determined two groups of research participants: a group of 32 patients aged 53–88 undergoing primary endovascular aneurysm repair and a control group of 43 volunteers aged 59–82 without the AAA. All the patients from the study group had their blood samples drawn in order to determine the level of calprotectin and RAGE in plasma. The second follow-up examination was carried out after three months. The concentration of calprotectin and RAGE in plasma was determined with the use of the immunoenzymatic method (ELISA). The study showed that patients with the AAA had significantly higher mean calprotectin and RAGE plasma levels (*p* = 0.0001 and *p* = 0.0002, respectively) as compared to the control group. After the AAA repair operations, the level of concentration of the calprotectin decreased significantly (*p* = 0.0002). So far, no studies on the connection between the increase of the calprotectin and RAGE in the patient’s plasma with the AAA have been published. Calprotectin may be a promising biomarker related to the occurrence of AAA. Larger studies are needed to fully elucidate and confirm the role of calprotectin in the development and progression of the aneurysm.

## 1. Introduction

Dilation of the abdominal aorta above 50% of the diameter of a healthy aorta is considered an abdominal aortic aneurysm (AAA). AAA is a complex, multifactorial disease with both genetic and environmental risk factors. The diameter of the AAA gradually increases and the larger ones are at higher risk of rupture. There are a lot of studied factors, which have impact on how fast the aneurysm grows, they comprise, amongst others: hypertension, smoking tobacco, hypercholesterolemia, genetic factors, chronic obstructive pulmonary disease, atherosclerosis as a general inflammatory process destructing aorta walls, and bacterial or viral infection [1,2]. 

Numerous biological factors have been identified that contribute to AAA occurrence, e.g., serum elastin peptides (SEP), plasmin-antiplasmin (PAP) complexes, metalloproteinase-2 (MMP-2), metalloproteinase-9 (MMP-9), P-elastase, cystein C, tissue plasminogen activator (tPA), interferon-gamma (IFN-gamma), tumor necrosis factor alpha (TNF-alpha), interleukin-8 (Il-8), interleukin-6 (Il-6), macrophage migration inhibitory factor (MIF), osteopontin, osteoprogerin, fibrynogen, homocysteine, *Staphylococcus sp*., *Chlamydophila pneumoniae*, *Treponema pallidum*, and arachidonic acid [3]. Moreover, in some cases, biomarkers such as arachidonic acid [4] and MMP-9 [5] have also been evaluated as indicators of possible success of the AAA therapy.

Recent studies show that calprotectin may be an important prognosis factor in cardiovascular and cardiometabolic diseases. Kruzliak et al. [6] found that calprotectin may be a useful marker in predicting the course of the atherosclerotic process. The proinflammatory protein calprotectin is connected with inflammatory arthropathy, vascular pathology, and acute coronary incidents. Angel et al. [7] proved that long-term therapy with TNF-α antagonists improved aortic stiffness and progression in carotid intima media thickness (CIMT) among patients with inflammatory arthropathies as well as they showed that calprotectin may be a soluble biomarker reflecting aortic stiffening in these group of patients.

In order to identify new biomarkers playing a significant role in etiopathogenesis of AAA, we took the calprotectin and the soluble eceptor for advanced glycation end products (sRAGE) into consideration. In the last few years, initial hypotheses were proposed on the basis of studies on animals and the connection between the biomarkers and extension of the abdominal aorta [8].

RAGE was first isolated from the surface of endothelium and described in 1992 [9]. The most pervasive circulating end products of advanced glycation (AGE), carboxymethyllysine (CML), reacts with RAGE on the first pro-inflammatory mediators by activating the nuclear factor-kappa B (NF-κB) [10]. Two mechanisms of producing sRAGE were identified: 1. transformation of full-length RAGE by proteasomes, such as RAGE cut by metalloproteinases (cRAGE); 2. mechanism by alternative burning at the level of RAGE gene expression, leading to an alternative secretion of the RAGE-endogenic mRNA (esRAGE) [11]. Research studies on AAA report similar impacts on the levels of MMP-9 induced by AGE/RAGE when macrophages were treated with sRAGE in order to stop RAGE signaling [12,13].

It is known that matrix metalloproteinases (MMP) are connected with etiopathogenesis of the AAA. AGE interact with cell receptors in order to increase cytokines release. Soluble receptors for AGE (sRAGE) and endogenic RAGE bind with AGE and reduce the release of cytokines [14]. 

A hypothesis was formulated that low levels of sRAGE would increase the level of cytokines, which would increase the level of MMP, thus contributing to the creation of an aortic aneurysm [11]. The end products of glycation are a non-uniform group of irreversible adjuvants, reacting with the AGE receptors (RAGE), increasing the gene expression, stimulating to produce inactive oxygen species (ROS) and release of cytokines. There are two isoforms of RAGE: completely soluble RAGE (sRAGE) and endogenic RAGE (esRAGE) [12,15,16]. 

Administration of sRAGE may act as a trap receptor for AGE and may inhibit binding AGE with RAGE, preventing development and progression of arteriosclerosis in animals. Moreover, AGEs/high mobility group box-1 (HMGB-1)—RAGE interaction is described as connected with heart failure and AAA [17].

Therefore, the purpose of this study was to investigate whether calprotectin and the RAGE plasma level may be a biomarker of human AAA occurrence. We assessed the correlation between the sRAGE with the diameter of the AAA and levels of calprotectin in plasma.

## 2. Material and Methods

### 2.1. Study Group

The study group comprised 32 patients hospitalized in the years 2017–2018 in the Vascular Surgery Department of the Regional Specialist Hospital in Wroclaw, Research and Development Centre, admitted for a planned operation of the AAA. The dilated aorta was diagnosed in an ultrasound examination and/or computed tomography of the abdomen of patients and finally confirmed during an operation. Patients were recruited after preparation of a protocol with specified significant elements determining elements qualifying persons for the study. Study inclusion criteria: aortic diameter > 30 mm, signed informed consent, age above 18; exclusion criteria: coexisting malignant disease and dialysis dependence. 

All patients had blood samples drawn at admission to determine the levels of the RAGE and calprotectin in plasma. After 3 months, patients had a control visit.

### 2.2. Control Group

The control group included 43 volunteers from the Regional Specialist Hospital in Wroclaw, Research and Development Centre. Each volunteer had an ultrasound examination confirming proper course of aorta and diameter of the vessel. Patients were recruited after preparation of a protocol with specified significant elements determining elements qualifying persons for the study. Study inclusion criteria: Caucasian race, age above 59, conscious written consent of a patient to be included in the study and have DNA isolated and stored, normal image of lower abdominal aorta. 

All the study participants read and signed a written informed consent for participation in the study. Due to the fact that sex is a risk factor of the AAA occurrence, the proportion between the number of men and women in line with the control group was maintained. 

### 2.3. Study Material

This research was approved by the Bioethics Commission at the Provincial Specialized Hospital in Wroclaw, Research and Development Centre (consent no. KB/6/2017). All participants gave written informed consent prior to participation and were of similar ethnic origin (Caucasian).

Both in the case of the study and the control groups, the peripheral blood, was drawn from the antecubital vein into probes with a proper anticoagulant. Patients from the study group had their blood samples drawn twice: before the operation and during a control check-up 3 months after the operation. For determination of calprotectin and receptors binding end products of glycation (RAGE), the blood was drawn into probes from EDTA (S-Monovette, 2.9 mL, Sarstedt). The probes were marked with a unique code and centrifuged at the speed of 2500 rpm, next thus obtained plasma was portioned and stored in −80 °C by the time of determination of concentrations. 

We determined the concentration of calprotectin and receptors binding end products of glycation (RAGE) in all persons included in the study and control group. The levels were determined in the Scientific Laboratory of the Regional Specialist Hospital in Wroclaw.

### 2.4. Study Methods

All patients taking part in the study were interviewed and qualified to the study. The concentration of calprotectin in plasma was determined with the use of the immune-enzyme method (ELISA) with the use of the Human Calprotectin AssayMax™ ELISA Kit (Assaypro, St. Charles, MO, USA). Human Calprotectin ELISA Kit, by a sandwich method with the use of polyclonal antibodies against human calprotectin and peroxidase enzyme. Receptors binding end products of glycation in plasma were marked with the immunoenzymatic method (ELISA) with the use of the Human Receptor for Advanced Glycation End Products (RAGE/AGER) ELISA Kit (Cusabio, Fannin St. Houston, TX, USA), by a sandwich method with the use of antibodies against receptors binding end products of glycation and horseradish peroxidase (HRP) enzyme. The whole procedure was carried out in accordance with the instructions for use attached by the manufacturer. The measurements were made with the use of the SPECTROstar Nano slides (BMG Labtech, Ortenberg, Germany), the measurement (BMG Labtech, Ortenberg, Germany).

### 2.5. Statistical Analysis

The mean concentrations of calprotectin and RAGE/AGER in the test group and in the control group were compared using the Mann-Whitney independent variable test. Calprotectin and RAGE/AGER concentrations in the study group before and after the procedure were compared using the Wilcoxon test. The statistically significant factor was established at *p* < 0.05. The PQStat package version 1.6.8 (Poznan, Poland) was used for statistical analysis.

## 3. Results

The study was conducted in patients diagnosed with an AAA by ultrasound examination and/or angio-CT (*n* = 32). All the diagnoses were confirmed by an intraoperative assessment. The mean maximum diameter of the aneurysm was 60.4 mm ± 15.03 mm (min: 39.0–max: 105.0 mm). The average diameter of the aorta in the control group was 19.9 mm ± 3.03 mm. Baseline characteristics of the participants are shown in Table 1. 

**Figure 1:**
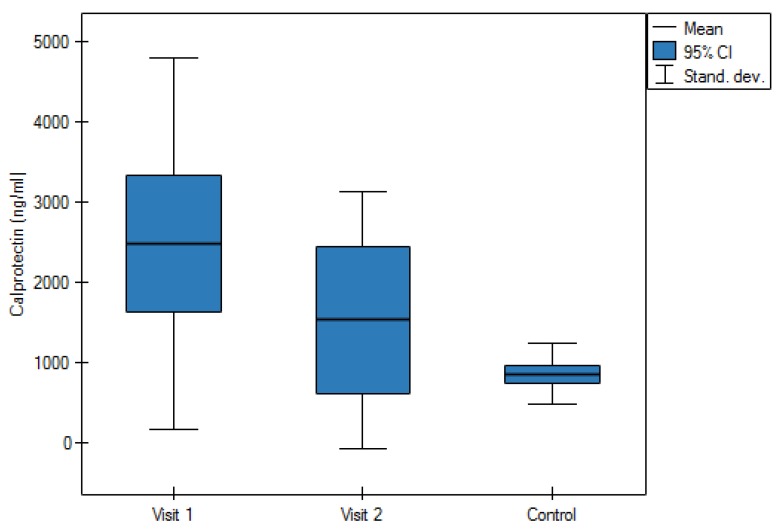
Average value of plasma concentration of calprotectin in the control group and study group during the first and second visit.

**Figure 2:**
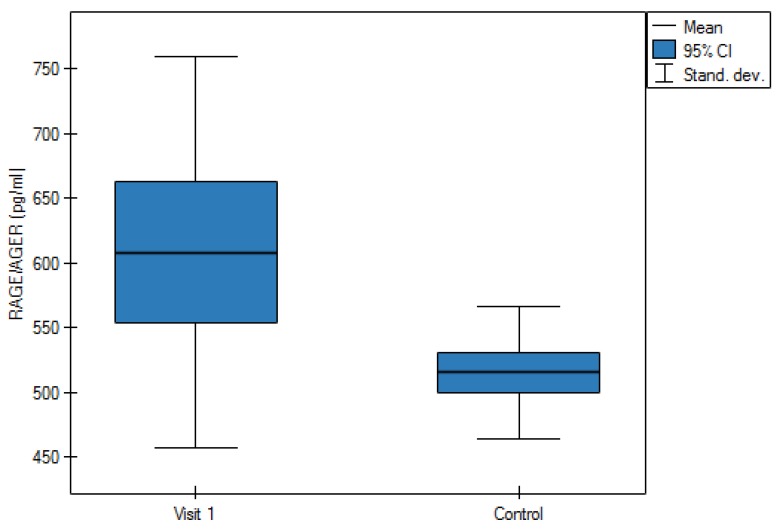
Average value of plasma concentration of receptor for advanced glycation end products (RAGE/AGER) in the control group and study group during the first and second visit.

The study carried out on a group of patients with AAA showed a significantly higher concentration of calprotectin and RAGE/AGER in the period prior to the operation as compared to the control group (*p* = 0.0001 and *p* = 0.0002, respectively). As showed in Table 2, the average value of concentration of calprotectin in plasma of the control group amounted to 861.4 ng/mL ± 378.4 ng/mL, and in the group with the AAA before the operation 2513.5 ± 2283.2 ng/mL and 1534.1 ng/mL ± 1600.3 ng/mL after the operation and at a follow-up accordingly (Figure 1). Average concentration of RAGE/AGER in the control group amounted to 515.3 ± 51.5 pg/mL and in the study group 608.4 ± 151.3 pg/mL prior to the operation and 657.4 ± 172.4 pg/mL a month after AAA repair operation (Figure 2). After AAA repair operations in patients, a decrease of concentrations of both studied factors was observed. Calprotectin level decrease after the surgery was statistically significant (*p* = 0.002).

The level of calprotectin and the RAGE receptor in the study group does not correlate with the size of the AAA.

## 4. Discussion

The study showed a significant correlation between an increase of the level of calprotectin and receptors binding end products of glycation (RAGE) and occurrence of the AAA. Similar results of the RAGE receptors in studies on animals showed [8]. Single studies on humans were carried out in the scope of RAGE or calprotectin [7,18]. Previous studies have found that the RAGE receptor and calprotectin play a significant role in cardiovascular diseases [19,20]. Thus far, there have been no studies published focusing on the connection between the increase of the level of calprotectin and the RAGE receptor in people with the AAA. Thus far, the calprotectin protein has not been examined in detail in terms of a correlation with the AAA, hence a limited number of studies on the issue is available.

The study did not show a statistically significant correlation between the increase of calprotectin and the RAGE receptor with the size of the AAA as in animals [8], thus this issue requires further studies. The studies showed a statistically significant increase of calprotectin and the RAGE receptor in patients with the AAA as compared to the control group. It seems that calprotectin is a substantially more sensitive marker than the RAGE receptor. The calprotectin showed even threefold increase in patients with the AAA as compared to the control group. In addition, almost a twofold decrease of the level of calprotectin after the operation of the AAA was observed as compared to the level of calprotectin prior to the operation.

In the control group, there was a proportion maintained between the number of men and women in line with the examined group, also with regard to the age, the control group corresponded to the study group. Each volunteer in the control group had an ultrasound examination confirming proper course of aorta and diameter of the vessel, which was not dilated due to the aneurysm. 

A threefold increase of the level of calprotectin in patients with the AAA, and a two-fold decrease of its level after the AAA repair operation shows the importance of the protein in etiopathogenesis of the AAA. 

Our findings suggest that calprotectin may be a promising biomarker for the occurrence of the AAA. The limitation of this study may be the small size of both patient and control groups, however, small studies avoid spending too many resources and financial costs to test the hypothesis. Therefore, data from this study should be used to design larger confirmatory studies. 

## 5. Conclusions

Patients with the AAA show a statistically significant increase of the concentration of calprotectin and receptors binding end products of glycation (RAGE) in their blood plasma. After the operation AAA, the concentration of calprotectin and receptors of end products of glycation marked a drop as compared to the reference group. To the best of our knowledge, this is the first report of calprotectin as AAA biomarker. It seems to be a promising biomarker related to the occurrence of AAA. Larger studies are needed to fully elucidate and confirm the role of calprotectin in the development and progression of AAA.

## Figures and Tables

**Table 1:** Characteristics of the control and study groups.

Characteristics	Control Group (*n* = 43) Mean ± SD (Range) or % of Patients	Study Group (*n* = 32) Mean ± SD (Range) or % of Patients
Age (years)	68.3 ± 6.4 (59–82)	70.3 ± 7.3 (53–88)
Sex (% of male)	93.0	93.7
Aorta /AAA diameter (mm)	19.9 ± 3.0 (12.9–24.2)	60.4 ± 15.0 (39–105)
Underlying disease		
Hypertension (%)	60.5	87.5
Diabetes mellitus type 2 (%)	21.9	14.0
Heart disease (%)	16.3	28.1
Tobacco smoke (%)	18.7	20.2

AAA, abdominal aortic aneurysm.

**Table 2:** Results obtained in the control and study groups.

	Study Group (*n* = 32) Mean ± SD	Control Group (*n* = 43) Mean ± SD
Age (year)	70.3 ± 7.26	68.3 ± 6.43
AAA/Aorta diameter (mm)	60.4 ± 15.03	19.9 ± 3.03
1st visit		
Calprotectin (ng/mL)	2513.5 ± 2283.2	861.4 ± 378.4
RAGE/AGER (pg/mL)	608.4 ± 151.3	515.3 ± 51.5
2nd visit (*n* = 14)		
Calprotectin (ng/mL)	1534.1 ± 1600.3	_
RAGE/AGER (pg/mL)	657.4 ± 172.4	_

AAA, abdominal aortic aneurysm; RAGE/AGER, human receptor for advanced glycation end products.

## Abbreviations

AAA	abdominal aortic aneurysm
CML	carboxymethyllysine
CIMT	carotid intima-media thickness test
ELISA	enzyme-linked immunosorbent assay
AGE	end products of advanced glycation
esRAGE	endogenous secretory receptor for advanced glycation end-products (esRAGE)
HMGB-1	high mobility group box-1
IFNγ	interferon-gamma
Il-6	interleukin-6
Il-8	interleukin-8
MIF	macrophage migration inhibitory factor
MMP-2	matrix metalloproteinase-2
MMP-2	matrix metalloproteinase-9
NF-κB	nuclear factor-kappa B
tPA	tissue plasminogen activator
PAP	plasmin-antiplasmin complexes
RAGE	receptor for advanced glycation end products
SEP	serum elastin peptides
sRAGE	soluble receptor for advanced glycation end products
TNF-α	tumor necrosis factor alpha
